# Occurrence of Wassermann Reaction in Hospital Patients

**Published:** 1916-04

**Authors:** 


**Occurrence of Wassermann Reaction in Hospital Patients.** A. A. Horner, Boston, Boston M. & S. Jour., Feb. 10, presents the following table:

| | Positive. | Negative. | Total. |
|---|---|---|---|
| All cases | 87 | 413 | 500 |
| Pneumonia | 10 | 49 | 59 |
| Emphysema | 2 | 6 | 8 |
| Pulmonary tuberculosis | 4 | 36 | 40 |
| Hemoptysis | 1 | 0 | 1 |
| Cardiac | | | |
| Auricular fibrillation | 2 | 0 | 2 |
| Aortic disease | 1 | 5 | 6 |
| Aortic and mitral disease | 1 | 5 | 6 |
| Mitral disease | 5 | 51 | 56 |
| Cardiorenal | 5 | 5 | 10 |
| Renal | 4 | 21 | 25 |
| Diabetes Mellitus | 2 | 6 | 8 |
| Erysipelas | 7 | 13 | 20 |
| Rheumatic fever | 2 | 16 | 18 |
| Other Arthritis | 2 | 9 | 11 |
| Typhoid | 5 | 14 | 19 |
| Influenza | 1 | 4 | 5 |
| Scarlet fever | 1 | 2 | 3 |
| Malaria | 1 | 3 | 4 |
| Sinusitis | 1 | 1 | 2 |
| Stomatitis | 1 | 1 | 2 |
| Brain tumor | 1 | 2 | 3 |
| Cirrhosis of liver | 2 | 6 | 8 |
| Anemia, primary | 1 | 1 | 2 |
| Cardiospasm | 1 | 0 | 1 |
| Peptic ulcer | 1 | 2 | 3 |
| Cancer (stomach) | 1 | 16 | 17 |
| Alcoholism | 1 | 30 | 31 |
| Heat Exhaustion | 1 | 1 | 2 |
| Syphilis | 18 | 2 | 20 |
| Acute Poisoning | 1 | 12 | 13 |
| Morphinism | 1 | 0 | 1 |
| Miscellaneous | 0 | 94 | 94 |

